# Cost Minimization Analysis of Same-Day Long-Acting Reversible Contraception for Adolescents

**DOI:** 10.1001/jamanetworkopen.2019.11063

**Published:** 2019-09-11

**Authors:** Tracey A. Wilkinson, Stephen M. Downs, Brownsyne Tucker Edmonds

**Affiliations:** 1 Children’s Health Services Research, Department of Pediatrics, Indiana University School of Medicine, Indianapolis; 2 Pediatric and Adolescent Comparative Effectiveness Research, Department of Obstetrics and Gynecology, Indiana University School of Medicine, Indianapolis

## Abstract

**Importance:**

Long-acting reversible contraception (LARC) is considered first-line contraception for adolescents but often requires multiple clinic visits to obtain.

**Objective:**

To analyze Indiana Medicaid’s cost savings associated with providing adolescents with same-day access to LARC.

**Design, Setting, and Participants:**

An economic evaluation of cost minimization from the payer’s (Medicaid) perspective was performed from August 2017 through August 2018. The cost model examined the anticipated outcome of providing LARC at the first visit compared with requiring a second visit for placement. The costs and probabilities of clinic visits, devices, device insertions and removals, unintended pregnancy, and births, according to previously published sources, were incorporated into the model. The participants were payers (Medicaid).

**Main Outcomes and Measures:**

The outcomes were the cost of same-day LARC placement vs LARC placement at a subsequent visit in US dollars, and rates of unintended pregnancy and abortion. One-way sensitivity analysis was done.

**Results:**

Same-day LARC placement was associated with lower overall costs ($2016 per patient over 1 year) compared with LARC placement at a subsequent visit ($4133 per patient over 1 year). Compared with the return-visit strategy, same-day LARC was associated with an unintended pregnancy rate of 14% vs 48% and an abortion rate of 4% vs 14%.

**Conclusions and Relevance:**

Providing same-day LARC could save costs for Medicaid, largely by preventing unintended pregnancy. Expected cost savings could be used to implement policies that make this strategy feasible in all clinical settings.

## Key Points

**Question:**

What are the cost savings, from the payer’s (insurance) perspective, associated with providing same-day access to long-acting reversible contraception (LARC) for adolescents?

**Findings:**

In this economic evaluation, same-day LARC placement was associated with overall lower costs ($2016 per patient over 1 year) compared with placement at a subsequent visit ($4133 per patient over 1 year). In addition, numbers of unintended pregnancies and abortions decreased in association with providing same-day LARC placement.

**Meaning:**

The findings suggest that providing same-day LARC placement may save payers money by preventing unintended pregnancy, and efforts to make this model of care feasible in all clinical settings should be undertaken.

## Teaser

This economic evaluation study uses a cost model to analyze Indiana Medicaid’s cost savings associated with providing adolescents with same-day access to long-acting reversible contraception compared with requiring a second visit for placement.

## Introduction

In the United States, nearly one-half (45%) of all pregnancies are unintended, and for adolescents, that number is closer to 85%.^1,2^ These rates are significantly higher than those in other developed nations with similar resources and are associated with an estimated cost of $21 billion to the US government each year.^2,3^ Given the known link between intended pregnancies and beneficial outcomes, the US Department of Health and Human Services has made one of the Healthy People 2020 Goals to increase the proportion of intended pregnancies by 10% between 2010 and 2020.^4^

Unintended pregnancy disproportionately affects women with lower incomes and those from racial and ethnic minority groups.^1^ In fact, the rate of unintended pregnancy for women with incomes below the federal poverty level is more than 5 times higher than the rate for women with incomes greater than 200% of the federal poverty level.^1^ Geography is also important to consider, because unintended pregnancy rates differ between and within states.^5,6^ This variability is often associated with differential access to care, especially primary care. Indiana, in particular, has both large geographic areas of low access to primary care and a lower ratio of physicians to population compared with the United States as a whole.^7,8,9^

Research^10,11^ has shown that, by removing barriers to access, effective hormonal contraception is more readily used by women, including adolescents, and leads to fewer unintended pregnancies and abortions. The key to these studies^10,11^ has been to provide no-cost, same-day access for women who need contraception, regardless of their chosen method. Compared with national statistics, the CHOICE Project^12^ (which provided no-cost, same-day access to contraception) was associated with a 79% reduction in teen pregnancy, an 80% reduction in teen births, and a 76% reduction in abortions.

With enhanced access, women of all ages chose and continued to use long-acting reversible contraception (LARC), such as intrauterine device (IUD) or arm implant, more than baseline rates.^10,11,12^ This form of contraception is important because it is not user dependent and provides very effective contraception (failure rate <1%).^13^ Recent analysis^14^ to examine the reasons for the decrease in adolescent pregnancy rates in the United States has found that the primary reason for this decrease is increased use of hormonal contraceptives. This supports the idea that efforts focused on improving access to and use of contraception will lead to improved reproductive health outcomes.

Despite the research showing the benefits of providing same-day access to all forms of contraception, in practice, clinics frequently require 2 visits to receive LARC. This 2-visit model is not a requirement of an insurance company but is a result of barriers within the clinical and billing systems that are compounded by the financial burden of stocking these expensive devices.^15,16^ The patient seeking contraception will have a first visit for counseling and, if LARC is chosen, a second visit must be scheduled to insert the LARC device. Unfortunately, when LARC is not offered the same day, women fail to return for the second visit for LARC placement more than one-half of the time.^17^ Among those who do return, many have engaged in unprotected intercourse in the intervening weeks.^18,19^ This barrier to access can be greater during the postpartum window and can increase the risk of a short interpregnancy interval (<18 months), which has known associations with adverse birth outcomes, such as preterm birth and low birth weight.^20,21,22^

Given the costs of unintended pregnancy and short interpregnancy intervals for Medicaid in particular, which serves a vulnerable population at particularly high risk for adverse pregnancy outcomes, we sought to examine the net cost of providing same-day LARC from a Medicaid payer perspective. The expected cost savings can provide a benchmark for potential investment in incentives to provide same-day LARC.

## Methods

The Indiana University institutional review board determined that this study did not need approval given the modeling methods and lack of human participants. This study follows the Consolidated Health Economic Evaluation Reporting Standards (CHEERS) reporting guideline.

To analyze the net costs of providing same-day LARC, we developed a decision model from the perspective of state Medicaid in Indiana. Our base case was a 16-year-old patient presenting for care and desiring LARC. To construct the model, we made certain simplifying assumptions (Box). In general, these assumptions tend to bias against same-day LARC because they ignore some costs of unintended pregnancy. Data analysis was performed from August 2017 through August 2018.

Box. **Study Assumptions**

When same-day LARC is available, whatever LARC method chosen would be available on the day of a visit.
If the patient does not return for LARC, she will not use another form of contraception.
There is no cost for pregnancy termination because it is not covered by Medicaid in most states.
Pregnancy outcomes are term delivery (vaginal or cesarean delivery), preterm delivery (vaginal or cesarean delivery), miscarriage, or termination.
Twin pregnancies are not modeled because of their infrequency.
Pregnancy complications are not modeled beyond the need for cesarean delivery.
Long-term health care (beyond neonatal care) costs for children are not included.

Abbreviation: LARC, long-acting reversible contraception.

### Decision Model

**Figure 1.**
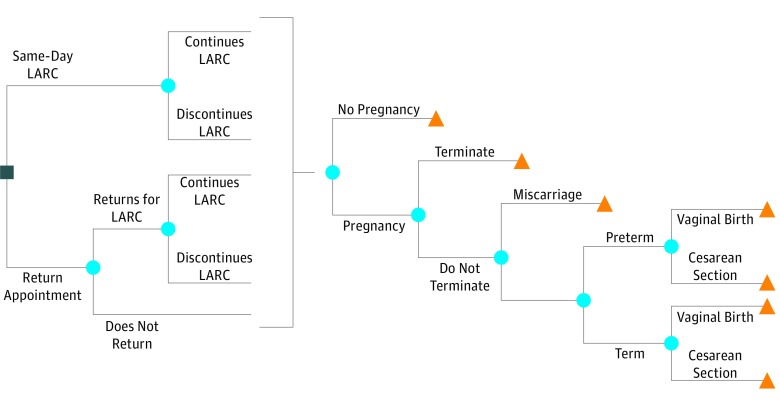
Long-Acting Reversible Contraception (LARC) Decision Tree The decision node (square) represents the options under evaluation. The chance nodes (circles) represent events that may happen. The terminal nodes (triangles) represent end points where costs are summed.

We constructed a decision tree (Figure 1) beginning with a decision node with 2 options: provide same-day LARC insertion or schedule a return appointment for LARC insertion. Following the option of scheduling a return visit for LARC insertion, a chance node models the possibility of the patient returning vs not. If the patient receives a LARC, she may choose to continue or have it removed.

After each of these events, the following events can happen. The woman may become pregnant or not. If she becomes pregnant, she might choose to terminate the pregnancy or not. If she does not terminate, she may have a miscarriage or not. If she retains the pregnancy, she will give birth preterm or at term, and in either case she may give birth vaginally or require a cesarean delivery.

### Probabilities and Costs

The probabilities at each chance node were estimated by review of the literature. The costs of each event were summed for each pathway in the decision tree. Probabilities and costs were estimated over a 1-year period. The probabilities and costs calculated using previously published sources^17,18,23,24,25,26,27,28,29,30,31,32,33^ are shown in the Table. The final expected cost for each option was calculated. In addition, we calculated the likelihood of pregnancy and pregnancy termination under each option.

**Table.** Baseline Costs and Probabilities

Costs and Probabilities	Baseline Value	Source	Threshold
Probabilities			
Probability that patient will continue using LARC	0.84	Diedrich et al,^24^ 2017	0.14
Probability of no pregnancy with LARC	0.99	Winner et al,^25^ 2012	0.28
Probability of pregnancy without contraception	0.85	Trussell et al,^26^ 2011	0.13
Probability patient will return for LARC insertion at a second visit	0.52	Bergin et al,^17^ 2012; Tocce et al,^18^ 2012	None
Probability of cesarean delivery	0.20	Martin et al,^27^ 2018	None
Probability of miscarriage	0.15	Sedgh et al,^28^ 2015; Kost et al,^29^ 2017	0.92
Probability of preterm delivery	0.13	Child Trends,^30^ 2015	None
Probability patient will terminate pregnancy	0.30	Sedgh et al,^28^ 2015; Kost et al,^29^ 2017	0.90
Costs (Medicaid reimbursement), $US			
Medicaid payment for maternal and newborn care after term delivery			
Vaginal	9131	Corry et al,^31^ 2013	None
Cesarean	13 590	Corry et al,^31^ 2013	None
Cost of			
Placing LARC^a^	74	Indiana Medicaid^32^	4692
LARC device^a^	776	Indiana Medicaid^32^	None
Miscarriage	644	Rausch et al,^33^ 2012	None
Prenatal care	750	Hueston et al,^23^ 2008; Indiana Medicaid^32^	None
Medicaid payment for			
Vaginal delivery and preterm newborn care	19 992	Corry et al,^31^ 2013	None
Maternal cesarean delivery and preterm newborn care	27 954	Corry et al,^31^ 2013	None
Cost of LARC removal^a^	90	Indiana Medicaid^32^	24 487

Abbreviation: LARC, long-acting reversible contraception.

^a^ Mean of intrauterine devices and contraceptive implant.

After building our decision tree, we consulted the peer-reviewed literature and government and institutional reports and used some state-specific information from the Indiana Office of Medicaid Policy and Planning for cost^32^ (ie, Medicaid reimbursement rates) and probability values for each variable (Table). For some variables, there were differences based on which type of LARC method was chosen. In these instances, we calculated a mean of those values.

A low-risk pregnancy carried to term includes 14 total prenatal visits. Although there has been a trend for more adolescents to establish prenatal care within the first trimester, approximately one-half of adolescents do not receive care until the second or third trimester.^23^ Therefore, we estimated an expected 10 prenatal visits in the adolescent population for the model.

For the probability that LARC will be continued, we used a rate for 1-year continuation of LARC from the CHOICE study.^12^ For the probability of returning for a second visit to obtain LARC placement, we looked to the literature^23,24,25,26,27,28,29,30,31,32,33^ and found rates from primary care as well as postpartum visits for LARC. We calculated a mean of these rates to account for the varied reasons a woman may present for same-day LARC in a clinic. To account for uncertainty in parameter estimates, we conducted 1-way sensitivity analysis on each variable (costs and probabilities) to determine the threshold value for each at which same-day LARC became more costly than scheduling a return visit.

## Results

At baseline, the overall cost of same-day LARC placement was $2016 per patient over a 1-year period. Scheduling a second visit for LARC placement was associated with an overall cost of $4133 per patient over the same 1-year period, $2117 more than providing LARC on the day requested. Same-day LARC was associated with an unintended pregnancy rate of 14% compared with a 48% rate for the return-visit strategy. Same-day LARC was associated with an abortion rate of 4% compared with 14% for the return-visit strategy.

**Figure 2.**
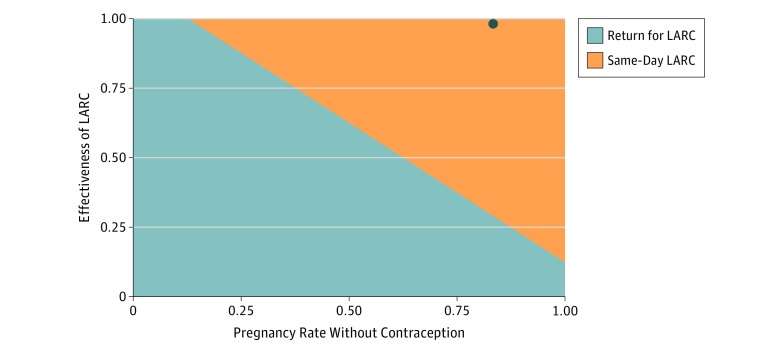
Sensitivity Analysis of Pregnancy Rate and Long-Acting Reversible Contraception (LARC) Effectiveness Graph shows 2-way sensitivity analysis showing all possible combinations of pregnancy rates without contraception and effectiveness estimates for LARC. The baseline values for these are indicated by the dot in the upper right corner. The threshold values are shown by the diagonal line between the area where LARC is associated with cost savings (orange) and where it is associated with increased costs (gray).

One-way sensitivity analysis to determine whether uncertainty in the variables would affect the outcomes showed that the model was sensitive to 5 probability estimates and 2 cost estimates (Table), meaning that beyond certain values for each of these variables, the 2-visit model would save money. However, these values were consistently very far from our baseline estimates. For example, if the probability that a patient would keep an inserted LARC device decreased from 81% to less than 14% or if the effectiveness of LARC decreased from 99% to 28%, the 2-visit process would be less expensive. The cost of placing LARC would have to increase from $74 to $4692 to offset the savings of same-day LARC. Likewise, the cost of removing a LARC device would have to increase from $94 to $24 487 before same-day LARC was more expensive. Figure 2 illustrates this with a 2-way sensitivity analysis showing all possible combinations of pregnancy rates without contraception and effectiveness estimates for LARC.

## Discussion

We sought to estimate the net cost of providing same-day LARC for adolescents from a payer’s (Medicaid) perspective. We found that, compared with a return-visit strategy, same-day LARC placement was associated with savings of more than $2000 per patient per year and with reduced unintended pregnancy and abortion rates. Previous literature has described the cost-effectiveness of LARC placement before postpartum hospital discharge, which led to policy changes in various states to separate insurance claims and enable placement of LARC before hospital discharge.^16,34^ To our knowledge, this is the first cost-minimization analysis done from the outpatient perspective. The cost savings calculated here provide a benchmark for policy makers to consider potential policy changes and investments in incentives to promote and provide same-day LARC.

Our findings add to a growing body literature in support of promoting, facilitating, and dissemination same-day LARC as the standard of care in policy and practice. In 2009, the American College of Obstetricians and Gynecologists published recommendations^35^ to adopt same-day LARC insertion protocols, indicating that clinicians need not await a certain time in the menstrual cycle, conduct screening for sexually transmitted infection, or await sexually transmitted infection test results to insert a LARC. Such delays, no longer accepted as an evidence-based standard of care, are now recognized as barriers to access rather than safeguards. Nevertheless, a 2013 study^36^ of family planning agencies in Colorado and Iowa found that only 18% of the agencies offered same-day IUD and 36% offered same-day implants, citing attitudinal and systemic barriers as preventing same-day offerings. In pediatric practices, even more training and infrastructural barriers exist, because many pediatric clinicians lack the training to place the devices and/or the equipment to perform speculum examinations in-office for IUD insertions.^37^ Barriers that cause delays for adolescent access to LARC are of particular importance. In a study of postpartum teens, delays for as little as a few weeks decreased the likelihood that young women would receive their intended LARC methods, and the group proved to be at particularly high risk for rapid repeat pregnancy.^18^

Furthermore, reimbursement policies and costs of providing same-day access to LARC represent large barriers to access.^16^ Despite efforts within the Affordable Care Act to ensure that contraceptives are part of all health insurance plans without cost sharing, same-day access is not always guaranteed. In fact, very few clinics that provide care to individuals with Medicaid or Medicaid expansion plans (ie, federally qualified health centers) are able to provide same-day access to all forms of contraception given the costs and staffing involved to make that feasible.^15^ Whether a LARC device is covered as a pharmacy or medical benefit also affects same-day access and can lead to delays in device reimbursement for clinics or an additional step of having to order from a pharmacy. Therefore, clinicians do not order them until after the patient has chosen this method because they do not want to incur the carrying cost of stocking LARC devices. Furthermore, once a device has been ordered for a particular patient, it cannot be billed under another patient’s name. Unused devices can be returned, but often that process includes an arduous amount of paperwork and handling fees.

These training, infrastructure, and reimbursement barriers present a critically important opportunity for health care professionals, public health practitioners, and policy makers to partner and develop strategies to ensure timely access and availability of LARC for women desiring these methods. Many of the financial barriers to same-day LARC could be overcome with a multipronged approach targeted at realigning financial incentives to encourage, and even reward, clinical practices that provide same-day LARC services. As an insurer to an at-risk, low-income population, covering almost one-half of the nation’s births, Medicaid has a financial interest in optimizing contraceptive access and reproductive health and is particularly well positioned to implement innovative strategies to incentivize and facilitate same-day contraceptive access. Such strategies might include the following. First, provide bonus payments for clinicians to incentivize same-day contraceptive access. Doing so would overcome the reimbursement-to-cost differential that leads to the 2-visit strategy and mitigate carrying-cost concerns. Notably, this incentive would have to be applicable to all types of contraception that are available same day in office, so as not to promote coercion. Second, create a single, uniform reimbursement structure, preferably as a medical benefit, to mitigate some of the procedural delays that occur when a device has to be ordered for an individual patient as opposed to being used for any presenting patient. Currently, practices do not know whether a given patient’s LARC will be covered as a pharmacy or medical benefit, which creates additional administrative burdens, prompting a follow-up visit to allow time for this added administrative effort. Third, pursue a strategy to purchase LARC devices in bulk and distribute devices up front to clinics desiring to provide same-day LARC access. In doing so, Medicaid would defray the carrying costs to clinics. These up-front costs could be recouped in cost savings in a short time, because delayed insertion costs almost twice as much as same-day insertion. Fourth, develop a policy whereby LARC devices that were ordered for a specific patient but ultimately unused after a certain time (eg, 90 days) could be used for another patient instead of having to order a new device and return the unused device.

### Limitations

Some limitations to our findings must be considered. First, the model may be oversimplified, mainly because we do not, for example, consider costs associated with children born from unintended pregnancy. Considering these costs would likely tend to favor same-day LARC. Furthermore, given our baseline assumptions, we were not able to account for women using a less-effective form of contraception if a LARC was not obtained or its use was discontinued. Second, because the model was developed from the payer’s perspective, we did not consider or include patient preferences or utilities in the model. Indeed, we assume that unintended pregnancy and abortion are undesirable outcomes, but we do not quantify those here or take them into account in the analysis. We have taken the simpler and more traditional approach of accounting for uncertainty in decision analytic models by using sensitivity analysis. However, when the model is so clearly insensitive to wide variations in the parameters, more elaborate approaches, such as probabilistic sensitivity analysis, do not generally yield additional insights. Third, we estimated the relative likelihood of women selecting IUDs vs implantable contraceptives and the number of prenatal visits by calculating a mean. However, give the model’s insensitivity to these variables, we think these approximations are unlikely to be important.

## Conclusions

This study contributes to the current literature informing efforts to advance reproductive health and access to family planning. Furthermore, our findings have implications for both policy and practice. Creating reimbursement strategies that incentivize same-day LARC insertion would likely save money. Further research and demonstration projects are needed to further explore the feasibility of instituting interventions such as incentive payments, reconfiguring reimbursement structures, or pursuing bulk purchasing strategies to promote and facilitate efforts to increase same-day LARC availability and improve access to contraceptive care.
